# Methicillin Resistant *Staphylococcus aureus* Prostatic Abscess with Bacteremia

**DOI:** 10.1155/2013/613961

**Published:** 2013-12-24

**Authors:** Daniel J. Lachant, Michael Apostolakos, Anthony Pietropaoli

**Affiliations:** ^1^Department of Medicine, University of Rochester Medical Center, 601 Elmwood Avenue, P.O. Box 692, Rochester, NY 14642, USA; ^2^Department of Pulmonary/Critical Care, University of Rochester Medical Center, 601 Elmwood Avenue, P.O. Box 692, Rochester, NY 14642, USA

## Abstract

Prostatic abscess is traditionally considered a rare disease that is caused by Gram-negative bacteria. Methicillin resistant *Staphylococcus aureus* (MRSA) has recently emerged as an important cause of prostatic abscesses. Symptoms are nonspecific and include dysuria, urinary frequency, fever, chills, and perineal and low back pain. Morbidity and mortality increase with delays in identification and proper treatment. We present two cases of community acquired MRSA prostatic abscesses with bacteremia. One of these cases may be the first reported septic shock fatality resulting from a prostatic abscess source in an immunocompetent patient. As the number of community acquired MRSA bacteremia cases increases, this potential site of infection should be recognized.

## 1. Introduction 

There is no clear consensus about the incidence of prostate abscess, but it is thought to be a rare but potentially fatal disease [1–6]. Typical symptoms include dysuria, frequency, fever, chills, and perineal and low back pain [7–9]. When clinically suspected, diagnostic workup includes cultures and imaging, and treatment includes broad spectrum antibiotics and drainage [4, 6–8, 10]. *Escherichia coli* and enteric organisms account for the majority of prostate infections. *Staphylococcus aureus* is reported much less commonly [1–3, 8–14], while methicillin resistant *Staphylococcus aureus* (MRSA) is considered rare [5]. Since 2000, multiple cases of hospital and community acquired MRSA prostatic abscess have been reported (Table 1) [1–6, 9–18].

We report two cases of community acquired MRSA prostatic abscess with bacteremia at the same medical center within a 2-year time interval. These cases are unique because they were young and healthy, without typical risk factors, and had particularly severe clinical illness. To our knowledge, the first case may be the first reported immunocompetent male dying of septic shock from a primary community acquired MRSA prostatic abscess.

*Case 1*. A 47-year-old male with past medical history of partial hypospadias with urethral stricture presented with a one-week history of diffuse myalgias, dry cough, anorexia, dyspnea, fever, and chills. He had been recently treated with penicillin for a presumed penile shaft furuncle. He was brought to the emergency department after becoming more somnolent and dyspneic. His wife reported that he had not complained of hematuria, abdominal pain, or dysuria. Upon presentation he was hypotensive and required intubation for respiratory failure. Vancomycin, piperacillin/tazobactam, stress dose hydrocortisone, and norepinephrine infusion were initiated. Despite maximal support, he expired 27 hours after admission. Negative diagnostic studies included *Clostridium difficile* toxin, nasal MRSA swab, nasal viral swab, sputum culture, influenza, respiratory syncytial virus, HIV 1/2, and *Legionella*. The two admission blood cultures and urine culture were positive for MRSA sensitive to clindamycin, gentamicin, linezolid, quinupristin/dalfopristin, tetracycline, trimethoprim/sulfamethoxazole, and vancomycin, with resistance to cefazolin, erythromycin, methicillin, oxacillin, and penicillin G.

**Figure 1:**
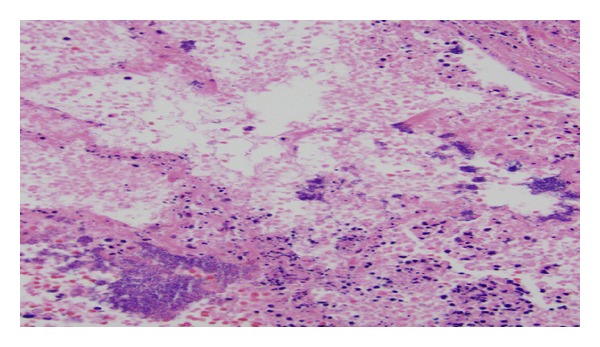
Alveolar tissue with septic emboli. Culture positive for MRSA.

**Figure 2:**
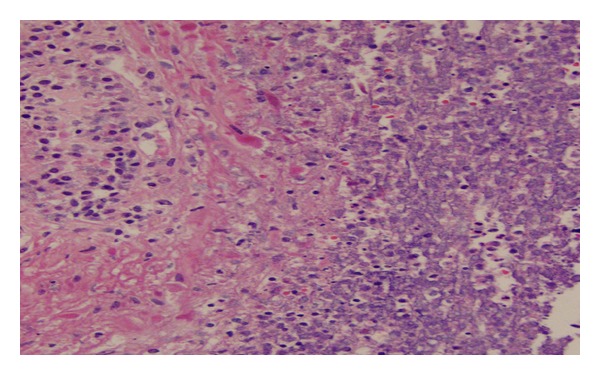
Prostatic tissue with polymorphonuclear cells. Culture positive for MRSA.

At the time of death, the working diagnosis was septic shock secondary to pneumonia. At autopsy, the lungs showed multiple septic pulmonary infarcts (Figure 1) with extensive bronchopneumonia. The mitral valve had 2 forms of bacterial vegetation. The spleen was enlarged (600 g) with a recent infarct. The right kidney also had an infarct and the left kidney had focal pyelonephritis. The prostate was asymmetric, larger on the left, with cavitations, necrotic tissue, and grossly purulent exudate (Figure 2). The prostatic venous plexus was thrombosed. The bladder did not have any pseudodiverticulosis or trabeculation and the ureters were unremarkable.

**Figure 3:**
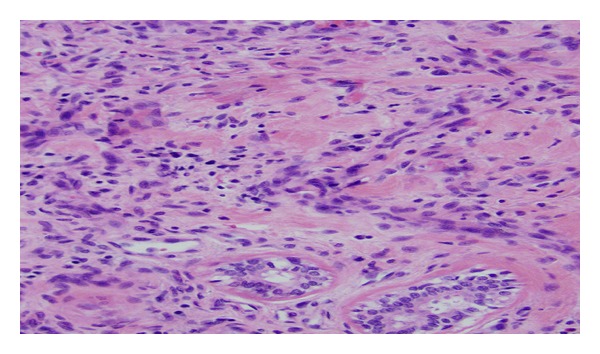
Penile lesion with chronic inflammatory cells. Culture negative for MRSA.

The final cause of death was septic shock from community acquired MRSA. The pathologist excluded the penile furuncle as the source of the MRSA bacteremia since only penile fibrous tissue was present (Figure 3), with no bacterial growth or suppurative fluid. The pathologist opined that the prostate gland was the primary source of bacteremia and metastatic infection of the lung, heart, and other organs. This opinion was based on histological evidence of more temporally advanced prostatic suppurative inflammation compared to the other affected organs. Postmortem bacterial cultures grew MRSA from the lung, heart, kidney, and prostate.

*Case 2*. A 31-year-old male with past medical history of sickle-beta thalassemia presented to the hospital with three days of left-sided pleuritic chest pain, fevers, rigors, and fatigue. He had received ciprofloxacin for complaints of dysuria 3 days previously. Physical examination showed a very tender prostate, clear lung sounds, and no cardiac murmurs. Urinalysis showed trace leukocyte esterase, 3 white blood cells, and 1 red blood cell. White blood cell count was 10,200 cells/Ul. A CT angiogram showed a patchy right middle lobe opacity and mediastinal adenopathy, without pulmonary emboli. He was treated with vancomycin and piperacillin/tazobactam. The two sets of blood cultures obtained on admission grew MRSA sensitive to clindamycin, gentamicin, linezolid, quinupristin/dalfopristin, tetracycline, trimethoprim/sulfamethoxazole, and vancomycin, with resistance to cefazolin, erythromycin, methicillin, oxacillin, and penicillin G.

**Figure 4:**
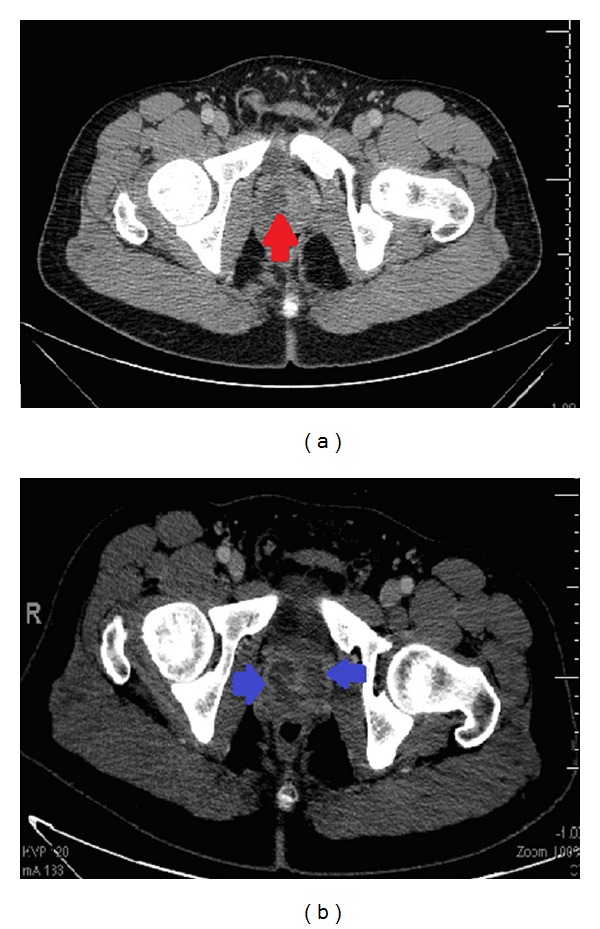
(a) Initial CT scan (left) showing 2.3 cm prostate abscess in the right gland (red arrow). (b) Repeat CT scan (right) 1 week later showed larger and multiple cystic fluid collections in the prostate gland (blue arrow).

His electrocardiogram suggested pericarditis, prompting an echocardiogram showing possible intra- and extramyocardial masses, no valvular vegetation, and a pericardial effusion without tamponade. Cardiac MRI showed minimal focal enhancement and a large exudative pericardial effusion with evidence of mild constriction. Pericardial fluid was not obtained. After what had been learned from the first case, a CT of the abdomen and pelvis revealed a 2.3 × 2.2 cm prostatic abscess (Figure 4). The abscess was transrectally drained and cultures grew MRSA. The patient was discharged home on intravenous vancomycin.

Three days later he again presented with fever, rigors, hematuria, pyuria, and left flank pain. A repeat CT showed worsening prostatic abscess (Figure 4). Blood cultures remained negative. Vancomycin was switched to daptomycin and a transurethral prostate resection (TURP) with abscess drainage was performed. His fever and other symptoms resolved and he completed an outpatient course of daptomycin and trimethoprim/sulfamethoxazole.

## 2. Discussion

Eighteen cases of hospital and community acquired MRSA related prostate infections have been reported worldwide, including our two cases from a single medical center (Table 1) [1–6, 9–18]. Of the total reported cases, the age ranges from 29 to 77 years. Sixteen of the patients had genitourinary (GU) complaints, seventeen had prostatic abscesses on imaging or autopsy, sixteen had bacteremia, ten had diabetes, and two had AIDS (Table 1) [1–6, 9–18]. The first reported fatal case was in a patient with AIDS [1], so to our knowledge our first patient is the only reported instance of an immunocompetent host succumbing to this infection.

There are no established treatment guidelines for prostatic abscess. Of the other reported cases, two were successfully treated with antibiotics alone, and the other fourteen required drainage and antibiotic regimens that included vancomycin, daptomycin, doxycycline, rifampin, sulfamethoxazole/trimethoprim, and nafcillin (Table 1) [1–6, 9–18]. Linezolid is an acceptable treatment option for MRSA prostate and urinary tract infections but was not used in any of these reported cases [19]. Our first case highlights the potential virulence of this infection and illustrates why early antibiotics and drainage are critical in severe cases.

Common risk factors and mechanisms for prostate infections include obstructive uropathy with retrograde flow of urine, straddle injury, urethral foreign bodies (e.g., chronic indwelling catheters and lower GU tract instrumentation), prostatitis, HIV infection, diabetes mellitus, immunodeficiency states, and bacteremia [4, 7, 11, 12, 14]. Our first patient had a urethral stricture, but autopsy showed no signs of obstructive changes in the bladder. We speculate that his history of a penile furuncle led to the prostatic abscess with subsequent development of acute bacterial endocarditis. The pathologist's temporal assessment favors this speculative conclusion. If correct, it is possible that earlier, more aggressive prostate abscess treatment may have been lifesaving. Despite the postmortem findings, we cannot rule out the possibility that the penile furuncle was the source of bloodstream infection and endocarditis, with subsequent metastatic prostatic infection.

Hematogenous seeding of the prostate was more likely in our second patient, who did not have any of the common prostatic abscess risk factors [11]. Once seeded with bacteria, prostatic abscess development is facilitated by diagnostic delay, impaired host defense [1], infection with organisms prone to abscess formation (e.g., *Staphylococcus aureus*) [2, 11], inadequate antimicrobial therapy [11], or poor antibiotic penetration into the prostate [5, 20].

Both hospital- and community-acquired MRSA variants are capable of infecting the prostate. In 2000, a new strain of community acquired MRSA, USA300 Panton-Valentine leukocidin (PVL) positive (MRSA 300), was reported. PVL is a potent toxin that confers greater virulence, increasing the likelihood of necrotizing pneumonia and greater complications of bacteremia including endocarditis, osteomyelitis, soft tissue infection, renal abscess, and now prostate abscess [10, 12, 15, 16]. MRSA 300 was identified to be the causative organism in two of the reported cases [15, 16]. We do not know if either of our patients had this MRSA strain.

Methicillin resistant *Staphylococcus aureus* is not commonly found in the urine with one study isolating 0.8% of 9,985 urine samples with MRSA [21]. Risk factors for MRSA in the urine, similar to prostate infections, include increased age, diabetes, hospital exposure, catheter use, genitourinary abnormalities, bacteremia, and pyelonephritis [21, 22]. Identification of MRSA in the urine should prompt a search for endovascular infection [22].

## 3. Conclusion

In summary, we present two cases of severe MRSA-induced prostatic abscess and review the published literature. We speculate that our first patient died from a prostatic abscess that subsequently seeded the bloodstream, causing acute bacterial endocarditis, widely metastatic infection, and septic shock. To our knowledge, this is the first reported case of fatal septic shock suspected to originate from a prostatic abscess in an immunocompetent host. As cases of MRSA bacteremia increase, physicians need to consider the prostate as a site of primary or persistent infection. The mainstays of treatment are early identification, appropriate antibiotics, and surgical drainage. Greater awareness of MRSA prostate infection should increase the likelihood that these treatments are promptly administered.

## Figures and Tables

**Table 1:** Clinical characteristics of patients with MRSA prostatic abscess.

Author	Age	Risk factors	Symptoms	Site of MRSA identification	Therapy	Outcome
Abreu et al. [6]	59	Diabetes	Yes	Blood	Vancomycin, ciprofloxacin, trimethoprim/sulfamethoxazole, and percutaneous drainage	Recovered
Baker et al. [11]	43	IVDA	Yes	Blood	Vancomycin, nafcillin, trimethoprim/sulfamethoxazole, and TURP	Recovered
Beckman and Edson [9]	53	Diabetes	Yes	Blood, nares	Vancomycin, trimethoprim/sulfamethoxazole, and rifampin	Recovered
Chao et al. [10]	40	AIDS (CD4 140)	Yes	Blood	Vancomycin, transperineal drainage	Recovered
Deshpande et al. [18]	49	BPH	Yes	None	Vancomycin, doxycycline, and TURP	Recovered
Flannery and Humphrey [14]	49	Diabetes	No	Blood, urine	Vancomycin, doxycycline, and TURP	Recovered
Fraser et el. [12]	63	Diabetes, scrotal abscess	Yes	Blood, urine, and scrotum	Vancomycin, interventional radiology drainage, and TURP	Recovered
Gautam et al. [1]	51	AIDS (CD4 135)	Yes	Blood	Vancomycin, ciprofloxacin, trimethoprim/sulfamethoxazole, and TURP	Died
Javeed et al. [16]	50	Diabetes	Yes	Blood, urine (USA300)	Vancomycin, daptomycin, and CT guided drainage	Recovered
Lachant et al. (Case 1 in current study)	47	Urethral stricture	No	Blood, urine, heart, and kidney	Vancomycin, piperacillin/tazobactam	Died
Lachant et al. (Case 2 in current study)	31	None	Yes	Blood	Vancomycin, piperacillin/tazobactam, daptomycin, trimethoprim/sulfamethoxazole, transrectal drainage, and TURP	Recovered
Lin et al. [15]	55	BPH	Yes	Blood (USA300)	Vancomycin	Recovered
Naboush et al. [17]	52	Diabetes	Yes	Blood, urine	Vancomycin, rifampin, trimethoprim/sulfamethoxazole, TURP	Recovered
Park et al. [2]	45	Diabetes	Yes	Blood	Vancomycin, TURP	Recovered
Pierce et al. [3]	64	Diabetes	Yes	Blood, urine	Vancomycin, percutaneous aspiration	Recovered
Shindel et al. [4]	29	Straddle injury with urethral stricture	Yes	Skin lesion	Vancomycin, transrectal cope loop catheter	Recovered
Sukhal et al. [5]	57	Diabetes	Yes	Blood, urine	Vancomycin	Recovered
Tobian and Ober [13]	56	Diabetes	Yes	Blood, urine, and right perinephric abscess	Vancomycin, rifampin, and transurethral unroofing	Recovered

TURP: transurethral resection of prostate.
